# Segmentation and Tracking of Lymphocytes Based on Modified Active Contour Models in Phase Contrast Microscopy Images

**DOI:** 10.1155/2015/693484

**Published:** 2015-05-18

**Authors:** Yali Huang, Zhiwen Liu

**Affiliations:** ^1^Institute of Signal and Image Processing, School of Information and Electronics, Beijing Institute of Technology (BIT), 5 South Zhongguancun Street, Haidian District, Beijing 100081, China; ^2^College of Electronics and Information Engineering, Hebei University, Baoding, China; ^3^Key Laboratory of Digital Medical Engineering of Hebei Province, Baoding, China

## Abstract

The paper proposes an improved active contour model for segmenting and tracking accurate boundaries of the single lymphocyte in phase-contrast microscopic images. Active contour models have been widely used in object segmentation and tracking. However, current external-force-inspired methods are weak at handling low-contrast edges and suffer from initialization sensitivity. In order to segment low-contrast boundaries, we combine the region information of the object, extracted by morphology gray-scale reconstruction, and the edge information, extracted by the Laplacian of Gaussian filter, to obtain an improved feature map to compute the external force field for the evolution of active contours. To alleviate initial location sensitivity, we set the initial contour close to the real boundaries by performing morphological image processing. The proposed method was tested on live lymphocyte images acquired through the phase-contrast microscope from the blood samples of mice, and comparative experimental results showed the advantages of the proposed method in terms of the accuracy and the speed. Tracking experiments showed that the proposed method can accurately segment and track lymphocyte boundaries in microscopic images over time even in the presence of low-contrast edges, which will provide a good prerequisite for the quantitative analysis of lymphocyte morphology and motility.

## 1. Introduction

The study of cell morphology and motility in microscopic images is essential to understand and treat various biological processes [1, 2]. As is well known, lymphocytes are involved in immune response. Clinicians observe that lymphocytes are highly deformable objects in special conditions, especially the graft rejection occurring. Quantitative analysis of lymphocyte morphology and motility is very meaningful in immune response research. The segmentation and tracking of lymphocyte boundaries is one of the prerequisites for the quantitative analysis of cell morphology and motility [3, 4]. Manual segmentation is subjective, time-consuming, and prone to errors. Therefore, automatic segmentation and tracking of cells are desired, and many such methods have been proposed over the years.

Traditional methods for image segmentation, such as thresholding, region growing, and watershed, could generate incorrect boundaries of objects since only local information is taken into consideration, while active contour models can segment, match, and track the object by exploiting (bottom-up) constraints derived from the image data together with (top-down) a priori knowledge about the location, the size, or the shape [5]. Hence they have been extensively studied and used in medical image segmentation ever since the introduction of active contour models in [6]. Zimmer et al. used parametric active contour models to segment and track migration cells in microscopic videos [7]. Acton's research group detected and tracked leukocytes based on the shape- and size-constrained active contour models [8, 9]. Meijering et al. applied the modified level set method to track cells with time-lapse fluorescence microscope [4]. Seroussi et al. used the directional gradient vector flow snakes to segment and track live cells in phase-contrast microscopic images [10].

Generally speaking, there are two types of active contours categorized by the representation and implementation: the parametric and the geometric active contours. The former type usually establishes energy function composed of internal and external energy terms. The latter is represented implicitly as the zero crossings of level set function, which can tackle topological changes elegantly at the cost of higher computational complexity [11]. Segmentation methods based on the parametric active contour models are used in our study since the observed lymphocytes are free of topological changes.

A number of methods for tracking cells have been developed over the past decades [4, 12]. In general, they can be divided into two categories according to the tracking strategies. The first type, based on the “first detect, then track” principle, initially detects the object in the first frame and then establishes the link between the detected objects from frame to frame based on certain criteria [13]. The second category of algorithms based on the integrated segmentation and tracking scheme is often referred to as active contour models. In active contour models, segmentation and tracking are performed simultaneously by fitting the model to the image data, and the result of the contour evolution in the previous frame is used as the initial contour of the subsequent frame. The advantage of these algorithms is that all available information from the previous frame can be directly incorporated into the segmentation of the subsequent frame. So tracking is realized by segmenting the object from frame to frame. In this paper, we adopt the second category of algorithms (active contour models) to segment and track cell boundaries.

Active contour models confront two challenges. First, inaccurate segmentations may occur when the edges are low-contrast or noise-contaminated. Second, active contour models are usually sensitive to the initial position. To address these difficulties, three types of methods have been proposed: edge-based models (where the energy optimization is driven by boundary information of the image), region-based models (where the energy optimization is driven by region information of the image), and hybrid models. Many external forces were proposed in different applications, such as balloons force [14], gradient vector flow (GVF) [15], virtual electric field [16], and external force using vector field convolution (VFC) [17]. The VFC snakes, within which an external force is computed by convolving the edge map with a user-defined vector field kernel, are more robust to noise and have less computational costs, compared with the classic GVF snakes. However, the initialization flexibility is still restricted. The initial contour, which was shown to be of importance [18, 19], evolves according to the external force field, and the external force field is computed via the edge map. The idea of the proposed external force field in this paper is inspired by [19], which introduced a modified feature map based on Harris detector for VFC snakes.

We note that the aforementioned methods of segmentation and tracking have focused on tracking the cell instead of the accurate extraction of cell boundaries. In order to segment and track the accurate boundaries of lymphocytes in the image sequence, we propose a novel feature map based on morphological gray-scale reconstruction and the LoG filter (MGRL) to compute external force field for the evolution of active contours. We make use of the region information of the given image by applying the morphological gray-scale reconstruction [20] and make use of the edge information through the LoG filter. To alleviate initialization sensitivity and reduce the number of iterations, the active contour is initialized close to the phase halo by an initial segmentation.

The rest of the paper is organized as follows.  Section 2 reviews several kinds of active contour models and their external force fields.  Section 3 proposes the improved external force based on MGRL-feature map, and the numerical implementation is given. The initialization of the lymphocyte contour is also introduced in this section. In Section 4, we apply active contour models with the proposed external force field to segment and track lymphocyte boundaries and compare experimental results with that of other methods. The conclusions are given in Section 5.

## 2. Related Work

Active contours are curves defined in an image domain that can move under the influence of internal forces and external forces [6]. Mathematically, the active contour is defined by a parametric contour *C*(*s*) = [*x*(*s*), *y*(*s*)], *s* ∈ [0,1] and evolves within the spatial domain of an image to minimize the following energy function:

(1) Esnakes=∫0112αC1s2+βC2s2+EextCsds,

where superscript “(*p*)” denotes the *p*th order derivative and *α* and *β* are weighting parameters representing the degrees of elasticity and rigidity of the active contour, respectively. The former makes the contour behave like an elastic string, while the latter makes the contour behave like a rigid rod [6]. The external energy *E*_*ext*_, which represents the image constraints, is defined to move the active contour toward an object boundary or other desired features. Using the calculus of variations [21], an active contour that minimizes (1) must satisfy the Euler equation

(2) $$\begin{array}{c} \alpha \cdot C^{\left(2\right)}\left(s\right)-\beta \cdot C^{\left(4\right)}\left(s\right)-\nabla E_{\mathrm{ext}}\left(C\left(s\right)\right)=0. \end{array}$$

The solution of (2) is obtained by calculating the steady state solution of the following gradient flow:

(3) $$\begin{array}{c} C_{t}\left(s,t\right)=\alpha \cdot C^{\left(2\right)}\left(s,t\right)-\beta \cdot C^{\left(4\right)}\left(s,t\right)-\nabla E_{\mathrm{ext}}\left(C\left(s,t\right)\right). \end{array}$$

Xu and Prince defined the gradient vector flow as the external force for the evolution of active contour [15]. They proposed the method by replacing −∇*E*_*ext*_(*C*(*s*)) in (2) with the vector field **V**(*x*, *y*) = [*V*_1_(*x*, *y*), *V*_2_(*x*, *y*)], which is computed by minimizing the function

(4) $$\begin{array}{c} E_{\mathrm{ext}}=\iint \mu \left({\left|\nabla V_{1}\right|}^{2}+{\left|\nabla V_{2}\right|}^{2}\right)+{\left|\nabla f\right|}^{2}{\left|V-\nabla f\right|}^{2}dx\;dy, \end{array}$$

where *μ* is a regularization parameter. One of the generally used forms of edge map *f* is

(5) $$\begin{array}{c} f\left(x,y\right)=\left|\nabla G_{\sigma }\left(x,y\right)*I\left(x,y\right)\right|, \end{array}$$

where *G*_*σ*_ is the Gaussian function with the standard deviation (STD)*σ* and *σ* = 3 in the study.

Li and Acton proposed another external force field for the evolution of active contours [17], in which the external force −∇*E*_*ext*_(*C*(*s*)) in (2) is replaced with the external force field **V**_vfc_(*x*, *y*):

(6) $$\begin{array}{c} V_{\text{vfc}}\left(x,y\right)=f\left(x,y\right)*k\left(x,y\right), \end{array}$$

where **k**(*x*, *y*) is the vector field kernel: $k(x,y)={\left(\sqrt{x^{2}+y^{2}}+\varepsilon \right)}^{-r}\cdot n(x,y)$; **n**(*x*, *y*) is the unit vector pointing to the kernel origin, $n(x,y)=[-x/\sqrt{x^{2}+y^{2}},-y/\sqrt{x^{2}+y^{2}}]$; *r* is a positive parameter to control the decrease, *r* = 2 in the study; and *ε* is a small positive number.

In active contour models, the external force, which is computed from the edge map, determines the evolution of the active contour. A good edge map (feature map) should emphasize the normal and the low-contrast edges equally. In order to reduce the effects of nonuniform edge intensities and highlight the low-contrast edges in phase contrast microscopic images, we propose a novel feature map instead of the traditional edge map to compute the external force, which is given as follows.

## 3. The Proposed Method

### 3.1. The Improved External Force Field Based on MGRL-Feature Map

The proposed feature map based on MGR and the LoG filter (MGRL-feature map) is

(7) $$\begin{array}{c} f_{\text{MGRL}}\left(x,y\right)=F_{l}\left[\nabla^{2}\left(G_{\sigma }\left(x,y\right)*\rho_{I\left(x,y\right)}\left(J\left(x,y\right)\right)\right)\right], \end{array}$$

where *F*_*l*_(·) is a low pass filter, which is realized by removing all connected region that have fewer than *P* (15~20) pixels from a binary image; ∇^2^(·) is the Laplacian operator; *G*_*σ*_(*x*, *y*)∗*ρ*_*I*(*x*,*y*)_(*J*(*x*, *y*)) is the convolution of the Gaussian function *G*_*σ*_(*x*, *y*) and *ρ*_*I*(*x*,*y*)_(*J*(*x*, *y*)).  *ρ*_*I*(*x*,*y*)_(*J*(*x*, *y*)) denotes the MGR of the *I*(*x*, *y*) from the *J*(*x*, *y*). *I*(*x*, *y*) is the region of interest (ROI). The MGR process can fill the “holes” induced by the intensity inhomogeneity in the image, which is defined as follows [20]. Let *J* and *I* be two gray-scale images defined on the same discrete domain *D*, where *J*(*p*), *I*(*p*)∈{0,1,…, *N* − 1} and *J*(*p*) ≤ *I*(*p*) for each pixel *p* ∈ *D*. The MGR *ρ*_*I*_(*J*) of *I* from *J* is given by *ρ*_*I*_(*J*)(*p*) = max⁡{*k* ∈ [0, *N* − 1]∣*p* ∈ *ρ*_*T*_*k*_(*I*)_(*T*_*k*_(*J*))}, ∀*p* ∈ *D*, where *T*_*k*_(·) is the successive thresholds; for *k* = 0,1,…, *N* − 1, *T*_*k*_(*I*) = {*p* ∈ *D*∣*I*(*p*) ≥ *k*}, these sets satisfy the following inclusion relationship *T*_*k*_(*I*)⊆*T*_*k*−1_(*I*), ∀*k* ∈ [1, *N* − 1].

We emphasize edges to look for zero crossings by filtering the MGR image *ρ*_*I*_(*J*) with the LoG filter. The STD of the LoG filter is 2, and the size is 13-by-13. Since the LoG filter detects many fragmentary edges simultaneously, we use a low pass filter *F*_*l*_(·) to omit the high frequency edges.

The external force field based on MGRL-feature map is

(8) $$\begin{array}{c} V_{\text{vfc}\_\text{MGRL}}\left(x,y\right)=f_{\text{MGRL}}\left(x,y\right)*k\left(x,y\right). \end{array}$$

**Figure 1:**
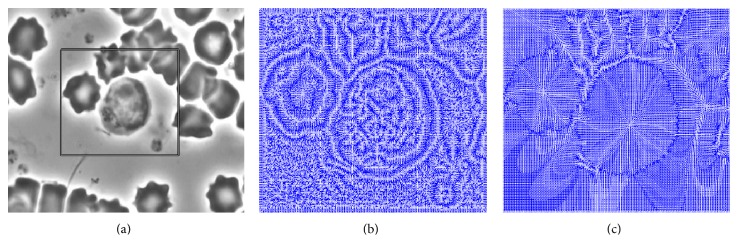
The original image and two differrent external force fields. (a) Cell image captured by the phase-contrast microscope, and the lymphoctye is in the center of the view. The ROI contains the target-lymphoctye, marked as in the rectangular. (b) The traditional external force field based on (5). (c) The proposed external force field based on (7).

In our study, phase contrast imaging is used for cell images acquisition in order to observe single lymphocyte morphology and motility over a long period of time. There is only one target-lymphocyte in the center of the view in each microscopic sequence, and the video is recorded by the help of the clinicians. An example frame is shown as Figure 1(a), in which the ROI containing the object-lymphocyte is marked as in the rectangular by the user. Two different external force fields of the marked rectangular are shown in Figures 1(b) and 1(c), respectively, which indicate that the external force field computed from the proposed feature map is sparser than that from the traditional edge map. Since the high frequency components of the feature map are removed by a low pass filter, the external force vectors only appear at the edges, which can accelerate the evolution of the active contour.

### 3.2. Numerical Implementation

The VFC snakes based on the improved external force minimize the following energy function:

(9) E=∫0112αC1s2+βC2s2+Evfc_MGRLCsds,

where *E*_vfc_MGRL_ denotes the improved external energy. Using the calculus of the variations [21], the minimization of (9) must satisfy the Euler equation

(10) $$\begin{array}{c} \alpha \cdot C^{\left(2\right)}\left(x,y\right)-\beta \cdot C^{\left(4\right)}\left(x,y\right)+V_{\text{vfc}\_\text{MGRL}}\left(x,y\right)=0, \end{array}$$

where **V**_vfc_MGRL_(*x*, *y*) = [*u*(*x*, *y*), *v*(*x*, *y*)] denotes the improved external force field derived from *E*_vfc_MGRL_. Equation (10) is equivalent to the following expression:

(11) αd2xds2−βd4xds4+ux,y=0,αd2yds2−βd4yds4+vx,y=0.

The solution of (11) is obtained by calculating the following gradient descent equation:

(12) xt=αx2−βx4+ux,y,yt=αy2−βy4+vx,y.

Using a finite difference approach on a discrete grid, our iterative solution to (12) is as follows [6]:

(13) xin+1=xin+γα·d2xinds2−β·d4xinds4+uxi,yi,yin+1=yin+γα·d2yinds2−β·d4yinds4+vxi,yi,

where *n* corresponds to discrete time and *γ* denotes the time step for each interaction. (*x*_*i*_, *y*_*i*_)  (*i* = 1,2,…, *N*) is the discrete approximation of (*x*(*s*_*i*_), *y*(*s*_*i*_)) by discretizing the interval $\left[\begin{array}{cc} 0 & 1 \end{array}\right]$ into *N* − 1 equispaced subintervals of length *h* = 1/(*N* − 1). Replace the derivative by the difference; then we get the second-order derivative of *x*(*s*), *d* ^2^ *x*(*s*)/*ds* ^2^ ≈ (*x*_*i*+1_ − 2*x*_*i*_ + *x*_*i*−1_)/*h* ^2^. The higher-order derivative can be obtained in this way.

### 3.3. Initial Contour

The active contour models may converge to an incorrect boundary if the initial contour is far from the real boundary. To alleviate the initialization sensitivity, we initialize the contour close to the real boundary by an initial segmentation. In the study, the initial contour of the object is determined by the following steps.

**Figure 2:**
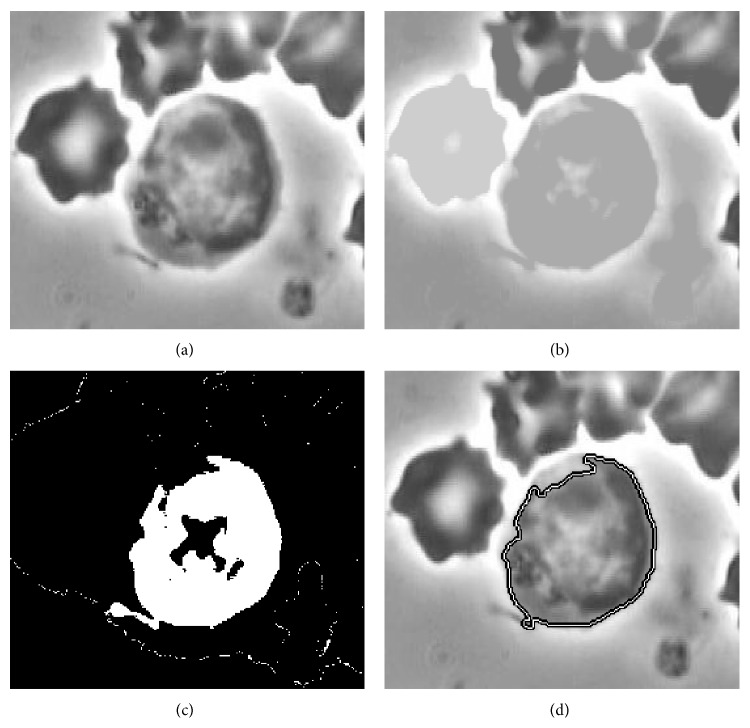
The procedure of obtaining the initial contour. (a) The ROI. (b) MGR of the ROI. (c) The coarse binary image of (b). (d) The initial contour extracted from the binary image.

(1) Select a rectangle ROI within the image by the user, as shown in Figure 2(a).
(2) Apply MGR to the ROI, as shown in Figure 2(b). The result shows that the lymphocyte region is distinct from other red, white cells and the background.
(3) Obtain the binary image from Figure 2(b) through thresholding techniques. The appropriate threshold is selected based on the intensity histogram distributions. The assumptions are as follows: there is only one target-lymphocyte in each image/frame, and there is no overlap occurrence between the target-lymphocyte and other cells. So the largest intensity distribution in the intensity histogram is selected as the optimal intensity threshold. Suppose the optimal threshold is thr; the image binarization processing is defined as (14). The binarized result is shown in Figure 2(c):
  (14) $$\begin{array}{c} B\left(x,y\right)=\left\{\begin{array}{cc} 1, & I\left(x,y\right)=\text{thr}\\ 0, & \text{others}. \end{array}\right. \end{array}$$
(4) After the binarization, morphological operations, including open and close, are applied to obtain the initial segmentation. The flat disk-shaped structuring element is used, and the specified radius is 3. And then we obtain the coarse binary image of the object. The initial contour, which is close to the real edge, is extracted from the binary image, as shown in Figure 2(d).

## 4. Experiments and Results

In the study, the image sequence of live cells were obtained by an optical phase-contrast microscope at a magnification of 16,000 from blood samples which were collected from the tails of mice (6–8 weeks old, 20–22 grams heavy). Note that in the experiments, there is only one target-lymphocyte in each image, which is at the center of the view and separate from other red and white cells.

### 4.1. Segmentation of Accurate Lymphocyte Boundaries

**Figure 3:**
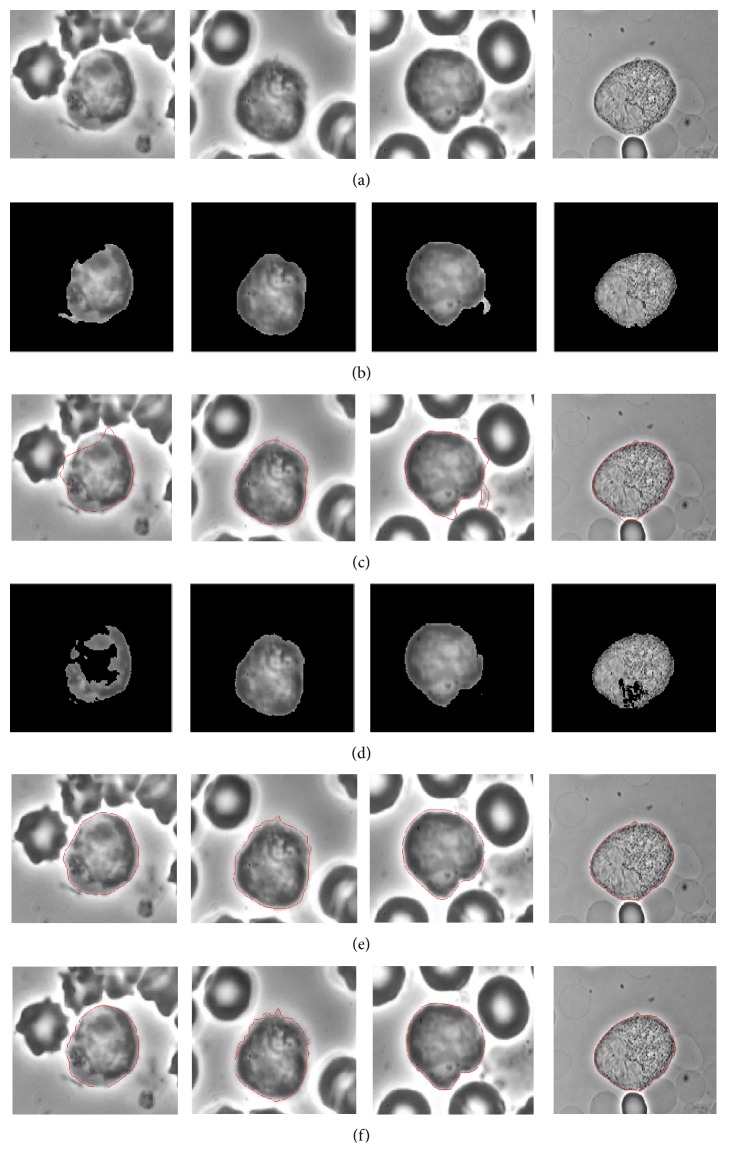
Segmentation of sample lymphocytes. The first line: the ROI (Img 1, Img 2, Img 3, and Img 4). The second, third, fourth, and fifth lines present the results for GAC, GVF, CV, and the proposed VFC_MGRL method. The last line is the ground truth.

We randomly selected 25 phase-contrast microscopic images. The result of manual segmentation is used as the ground truth in many studies although they have limitations. Therefore the average of the manual segmentation results by three experts is used as the ground truth in our study. To validate the performance the proposed method, we compared segmentation results with the other three types of widely used active contours: GVF snakes, edge-based geodesic active contour (GAC), region-based Chan, and Vese active contour model (CV). In the validation of GVF snakes, the active contour evolution equation is similar to (13), except that it uses gradient vector field as the external force. The evolution of GAC is ∂*ϕ*/∂*t* = *g* · (*k* + *c*)|∇*ϕ*| + ∇*g* · ∇*ϕ*, where *c* is the balloon force, *k* is the curvature, and *g*(·) is the edge function. The evolution of CV is ∂*ϕ*/∂*t* = *δ*(*ϕ*)[*μ*_1_ · *k* − *ν* − *λ*_1_(*I* − *c*_1_)^2^ + *λ*_2_(*I* − *c*_2_)^2^], where *c*_1_ and *c*_2_ are two constants which are the average intensities inside and outside the contour, respectively; *μ*_1_ ≥ 0, *ν* ≥ 0, *λ*_1_ > 0, *λ*_2_ > 0 are fixed parameters. The detailed parameters settings of the four methods (GVF, GAC, CV, and the improved VFC) are listed in Table 1.  Figure 3 shows the comparison among GAC, GVF, CV, the proposed method, and the manual segmentation results. The initial contours of the active contour models were obtained by the initial segmentation as mentioned above (see Section 3.3). The iteration numbers and the execution time are shown in Table 1. The experiments were conducted on a 2.93 GHz CPU, 4.00 G RAM computer. As we can see, the results of GVF and GAC suffered from the edge leaks outside of cells due to the low-contrast boundaries. The CV method failed to segment cell images with intensity variation inside the cell. The proposed method VFC_MGRL can find the accurate boundaries at low-contrast edges due to the improved external force field. The segmentation result of the proposed method is close to the expert's manual segmentation result, even better in the details. For manual segmentation by the expert, it is hard to segment the cellular protrusion consistently, but the proposed method can converge to the details of the edges.

**Figure 4:**
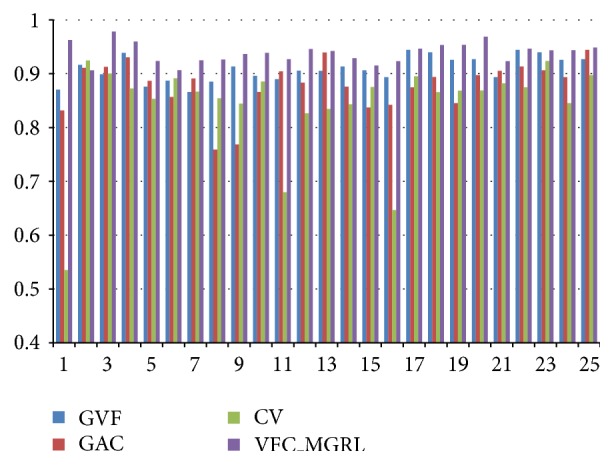
Detailed evaluation results. Horizontal axis shows the numbered images used for evaluation. Separate bars indicate the results of different methods: blue (the first bar) is GVF; red (the second bar) is GAC; green (the third bar) is CV; purple (the fourth bar) is the proposed VFC_MGRL method.

To quantitatively evaluate the segmentation, the Jaccard coefficient (JC) is employed to measure the similarity between the segmentation result and the ground truth, which is defined as the ratio between the size of the intersection of the sets and the size of their union; namely, JC = (*A*∩*B*)/(*A* ∪ *B*), where *A* denotes the segmentation result and *B* is the ground truth.  Figure 4 shows the detailed evaluation results for the 25 images separately. Parameters settings are shown in Table 1. It is important to note that the proposed VFC_MGRL snakes method outperform their traditional counterparts in most cases.

To compare the performance of different segmentation methods, the statistical analysis of segmentation results by different methods are shown in Table 2, in which STD means standard deviation.

### 4.2. Tracking of Accurate Lymphocyte Boundaries

**Figure 5:**
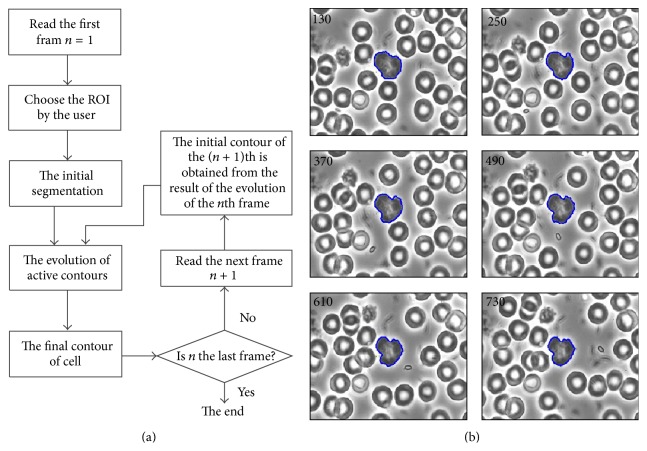
(a) The workflow of segmentation and tracking of the target-cell in an image sequence. (b) Segmentation and tracking of cell by using VFC_MGRL snakes at frame 130, 250, 370, 490, 610, and 730, respectively. The frame interval is 0.04 seconds.

Active contour models allow us to solve both segmentation and tracking problems simultaneously. The concept is that lymphocyte tracking is realized by lymphocyte segmentation frame by frame. The initial contour of the lymphocyte in the first frame is obtained by an initial segmentation, and for the second frame, the initial contour is obtained from the result of the contour evolution in the previous frame. That is to say, the final contour of the previous frame is regarded as the initial contour of the current frame during the evolution of active contours. The procedure of segmentation and tracking is described in Figure 5(a).

**Step 1 .**

Read the first frame of the video, and then choose the ROI containing the target-lymphocyte by the user, as shown in Figure 1(a).

**Step 2 .**

Extract the initial contour close to the ground truth. In the first frame of the video, this is realized by MGR and thresholding techniques (as introduced in Section 3.3); in the subsequent frames, the initial contour is obtained from the previous frame since the vibration of the lymphocyte position is not distinct between the successive frames.

**Step 3 .**

Compute the improved external force field of the ROI, and then implement the evolution of active contours according to the initial contour. The result of the evolution of the active contour is the final boundary of the lymphocyte.

**Step 4 .**

If the current frame is the last frame, the lymphocyte tracking procedure is over; if not, go to the next frame of the video repeating Step 3 till the last frame.

The accurate boundary of the lymphocyte is then obtained from frame to frame by VFC_MGRL snakes.

One result of lymphocyte tracking was shown in Figure 5(b), which shows that the proposed algorithm can accurately track the target-lymphocyte boundaries and follow the dynamic change of lymphocyte shape over time in a semiautomated fashion. In the first frame, the ROI was chosen by the user; in the subsequent frames, the object boundaries were segmented and tracked automatically.

## 5. Conclusions

The paper proposes a VFC_MGRL active contour model for segmenting and tracking of accurate boundaries of the single lymphocyte in phase contrast microscopic images. In a video, starting from the initial contour, the active contour converges to the accurate boundary according to the improved external force field. The MGRL-feature map can make full use of the given image by incorporating the advantages of MGR and the LoG filter. Therefore, it can be used for defining an efficient external force field when detecting low-contrast boundaries. To alleviate initialization sensitivity problem, the initial contour of the first frame is abstracted around the ground truth by an initial segmentation; the initial contour of the subsequent frame is obtained from the previous frame. The approach is tested on phase contrast microscopic images and performs better than other methods, which will provide a good prerequisite for the quantitative analysis of lymphocyte morphology and motility.

## Figures and Tables

**Table 1:** Parameters settings and execution times for images 1–4 of the four kinds of active contour methods.

	Parameters settings	Number of iterations	Run time (seconds)			
			Image 1	Image 2	Image 3	Image 4
GVF	*r* = 1, *α* = 0.8, *β* = 0.2, *μ* = 0.1	100	2.26	2.25	2.39	2.17
GAC	*dt* = 0.1, *c* = 1	200	4.08	3.99	4.16	4.05
CV	*dt* = 0.1, *μ*_1_ = 0.2, *ν* = 0, *λ*_1_ = *λ*_2_ = 1	200	2.92	2.63	2.63	2.72
VFC_MGRL	*r* = 1, *α* = 2, *β* = 0.2, log⁡filter : sigma = 2, size = 13	100	1.71	1.78	2.05	1.91

**Table 2:** The mean and standard deviation of JC by different segmentation methods.

	GVF	GAC	CV	VFC_MGRL
Mean	0.9091	0.8787	0.8422	0.9388
STD	0.0235	0.0460	0.0898	0.0185
